# Variation of Macro- and Microelements, and Trace Metals in Spring Wheat Genetic Resources in Siberia

**DOI:** 10.3390/plants11020149

**Published:** 2022-01-06

**Authors:** Sergey Shepelev, Alexey Morgounov, Paulina Flis, Hamit Koksel, Huihui Li, Timur Savin, Ram Sharma, Jingxin Wang, Vladimir Shamanin

**Affiliations:** 1 Laboratory of Grains Quality, Omsk State Agrarian University, 644 008 Omsk, Russia; ss.shepelev@omgau.org (S.S.); hamitkoksel@gmail.com (H.K.); 2 Saudi Arabia Country Office, Food and Agriculture Organization of the United Nations, Riyadh 11421, Saudi Arabia; alexey.morgounov@gmail.com; 3 Future Food Beacon of Excellence and the School of Biosciences, University of Nottingham, Nottingham LE12 5RD, UK; paulina.flis@nottingham.ac.uk; 4 Nutrition and Dietetics Department, Istiniye University, Istanbul 34010, Turkey; 5 CIMMYT-China, Institute of Crop Sciences, Chinese Academy of Agricultural Sciences, Beijing 100081, China; lihuihui@caas.cn (H.L.); rachaelxxxx@icloud.com (J.W.); 6 Department of Science, S. Seifullin Kazakh Agro Technical University, Nur-Sultan 010 000, Kazakhstan; savintimur_83@mail.ru; 7 International Center for Agricultural Research in Dry Areas, Central Asia and the Caucasus Regional Program, Tashkent 100 084, Uzbekistan; rcsharma20@outlook.com

**Keywords:** biofortification, cereals, nutritional quality, protein concentration

## Abstract

Western Siberia is one of the major spring wheat regions of Russia, cultivating over 7 Mha. The objective of the study was to evaluate the variation of macro- and microelements, and of trace metals in four distinct groups of genetic resources: primary synthetics from CIMMYT (37 entries), primary synthetics from Japan (8), US hard red spring wheat cultivars (14), and material from the Kazakhstan–Siberian Network on Spring Wheat Improvement (KASIB) (74). The experiment was conducted at Omsk State Agrarian University, using a random complete block design with four replicates in 2017 and 2018. Concentrations of 15 elements were included in the analysis: macroelements, Ca, K, Mg, P, and S; microelements, Fe, Cu, Mn, and Zn; toxic trace elements, Cd, Co, Ni; and trace elements, Mo, Rb, and Sr. Protein content was found to be positively correlated with the concentrations of 11 of the elements in one or both years. Multiple regression was used to adjust the concentration of each element, based on significant correlations with agronomic traits and macroelements. All 15 elements were evaluated for their suitability for genetic enhancement, considering phenotypic variation, their share of the genetic component in this variation, as well as the dependence of the element concentration on other traits. Three trace elements (Sr, Mo, and Co) were identified as traits that were relatively easy to enhance through breeding. These were followed by Ca, Cd, Rb, and K. The important biofortification elements Mn and Zn were among the traits that were difficult to enhance genetically. The CIMMYT and Japanese synthetics had significantly higher concentrations of K and Sr, compared to the local check. The Japanese synthetics also had the highest concentrations of Ca, S, Cd, and Mo. The US cultivars had concentrations of Ca as high as the Japanese synthetics, and the highest concentrations of Mg and Fe. KASIB’s germplasm had near-average values for most elements. Superior germplasm, with high macro- and microelement concentrations and low trace-element concentrations, was found in all groups of material included.

## 1. Introduction

Spring wheat is an important crop in Russia, with an annual cropping area of 11–12 Mha. The main spring wheat-production belt stretches from the mid-Volga region across the southern Ural mountains to Western Siberia. This is a short-season crop grown from May to August in an extensive, rainfed cropping system dominated by cereals, and occasionally rotated with oilseed and legume crops. Morgounov et al. [1] compared the effects of climate change on spring wheat production in Eurasia (Russia and Kazakhstan) and North America. The grain yields and production gains in North America were almost twice that in Eurasia, owing to better environmental conditions, including precipitation, as well as application of more advanced and diverse production technologies based on conservation agriculture. Wheat produced in Russia is traded both regionally and internationally. According to the FAO (www.fao.org/faostat, accessed on 1 January 2019), Russia exported 43.9 Mt of wheat grain in 2018. Therefore, grain quality, including health benefits or hazards, is important for global food security and safety.

The ionome has been defined as the mineral element composition of an organism, and represents the inorganic component of cellular and organismal systems [2]. Therefore, ionomics involves the quantitative measurement of the element composition of living organisms, and of changes in this composition in response to environment, growth stage, and genotype. Minerals comprising wheat grain can be divided into three groups: macroelements (Ca, K, Mg, P, Cl, and S) that are important for starch and protein formation; toxic heavy metals (arsenic (As), cadmium (Cd), chromium (Cr), and lead (Pb)), normally regulated not to exceed certain concentrations; and microelements essential for plants and humans (selenium (Se), boron (B), Mn, Cu, Fe, Mo, and Zn), which can also be harmful when exceeding certain concentrations. All five microelements, along with Ca and iodine, were selected as candidates for biofortification to improve nutritional value of crops, including wheat [3].

Wheat biofortification has been successfully applied for increasing Zn content in grain with commercial cultivars being grown in India and Pakistan [4,5]. A nutrition study with preschool children and women showed that consumption of high Zn-biofortified wheat prevented morbidity [6]. The concept of biofortification has not been applied in wheat breeding in Russia, and only a few studies have assessed the mineral composition of wheat cultivars grown in Russia. Bityutskii et al. [7] studied around 30 registered wheat cultivars and found variation in the microelement concentrations of grains (µg/g), Fe 15–22, Zn 14–21, and Mn 2.4–4.1. Morgounov et al. [8] determined 15 macro- and microelement concentrations in 49 genotypes grown across six sites in Russia and Kazakhstan in two years. Several cultivars (Element-22, Lutescens-3-04-21-11, and Silach) were identified as having high grain yields, relatively high protein content, and high concentrations of P, S, Mn, Cu, and Zn, singly or in combination. The above studies suggest that it could be possible to develop a varietal development program for improving mineral nutrients. In particular, the presence of multiple elements in combination in the same genotype indicates a positive association among them. A positive correlation between two elements could be exploited in a breeding program more efficiently, and could simultaneously improve them, using a selection index [9].

The success of wheat biofortification at CIMMYT was largely based on the utilization of genetic resources with high concentrations of nutritionally valuable elements [10]. Significant genetic variation for grain Zn and Fe was found in landraces and ancestors of common wheat, such as *Aegilops tauschii*, *Triticum turgidum* ssp. *dicoccoides*, *T. turgidum* ssp. *Dicoccum*, and *T. aestivum* ssp. *spelta*. Einkorn (*Triticum monococcum*) was later added to this list [11]. This variation has been introduced into high-yielding germplasm through conventional breeding and marker-assisted breeding [5].

Western Siberia (Kurgan, Tuymen, Omsk, Novovsibirsk, and Altai regions) grows around 7 Mha of spring wheat with an average grain yield of 2 t/ha. Drought is the most common abiotic stress, and leaf and stem rust are the main diseases affecting the crop. Spring wheat breeding programs in the region are united through the Kazakhstan–Siberian Network on Spring Wheat Improvement (KASIB). This network was established in 2000 with the objective of exchanging germplasm and conducting cooperative multilocational testing to characterize advanced breeding lines and new cultivars. The majority of the cultivars grown in the region represent tall, daylength-sensitive material with good drought tolerance and suitable bread-making quality [12]. The studies of KASIB germplasm demonstrated limited genetic diversity for resistance to stem [13] and leaf rust [14]. Overall, cultivars grown in the region have similar genetic makeup and high phenotypic similarity.

In order to expand spring wheat’s diversity, a panel of genetic resources (OMON-GAI (Omsk Observation Nursery–Genetically Associated Improvement)) was assembled at Omsk State Agrarian University, the KASIB network coordinator for Russia. The panel included primary synthetics from CIMMYT and Japan, cultivars from USA, Omsk cultivars developed by the university, and Omsk Agrarian Research Center and the KASIB network’s germplasm. The panel was phenotyped for common agronomic traits in 2017–2020 and genotyped using the genotyping-by-sequencing method, resulting in over 46,000 SNPs (single nucleotide polymorphism). The genetic diversity study clearly separated all material into three groups: CIMMYT synthetics, Japanese synthetics, and a combined group of bread wheat germplasm from KASIB and the USA [15]. A genome-wide association study (GWAS) was conducted on yield and 26 yield-related traits, disease resistance, and grain quality traits [16]. The study identified 243 significant marker–trait associations for 35 traits that explained up to 25% of the phenotypic variance, with the most significant of these having already been used in the marker-assisted breeding at the university.

Ionome phenotyping of the OMON-GAI panel for 23 elements was performed using grain from the 2017 and 2018 seasons. The objective of the study was to evaluate the variation of macro- and microelements, and trace metals in the different groups of genetic resources, identify the relationship between the agronomic and quality traits and element concentrations, select superior genotypes, and develop approaches to be used for breeding to enhance the element composition of wheat cultivars.

## 2. Results

### 2.1. Variation for Agronomic Traits

The analysis of variance of the number of days to heading, TKW, grain yield, and protein content data demonstrated the high significance of genotypes, years, and their interaction, except for the effect of years on TKW (Table S1). The weather conditions during the spring wheat growing season in 2017 were characterized by air temperatures close to the long-term average (17.3 °C in May–August versus 17.0 °C) and a moderate moisture stress, with precipitation of 163 mm during May–August, compared to the 238 mm long-term average. The 2018 growing season was cooler (15.2 °C in May–August) with 270 mm of rainfall. Leaf and stem rust affected susceptible genotypes with up to 30–40% severity in 2017 and up to 60–80% in 2018. These pathogens certainly affected the grain yield. The average yield of the whole panel was 320 g/m^2^ in 2017 and 395 g/m^2^ in 2018.

**Figure 1:**
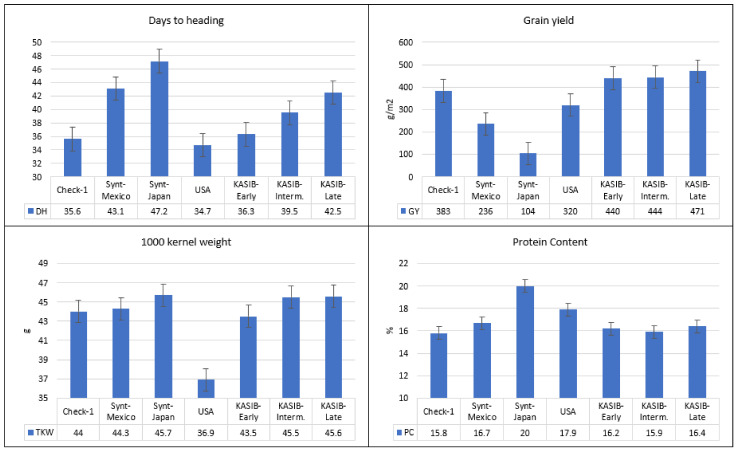
Variation for agronomic traits in different groups of genetic resources, average values for 2017–2018, and bars representing standard error.

The relative performance of the different groups of genetic resources across the two years is presented in Figure 1 and for individual years in Table S2. The number of days to heading varied from 35 (USA group) to 47 days (Japanese synthetics). The differences between the three KASIB groups were 36.3 days for the early group, 39.5 for the intermediate, and 42.5 for the late group. The highest grain yield was recorded for the three KASIB groups (440–471 g/m^2^), followed by the US cultivars (320 g/m^2^), the CIMMYT synthetics (236 g/m^2^), and the Japanese synthetics (104 g/m^2^). The early maturing check Pamyati Azieva demonstrated a grain yield of 399 g/m^2^, and the intermediate-maturing check Serebristaya, 470 g/m^2^. TKW was in a range of 44.0–45.7 g for all groups, though the US cultivars had smaller grain (36.9 g). The highest protein content was recorded for the Japan synthetics (20%), followed by the US cultivars (17.9%), the CIMMYT synthetics (16.7%), and the KASIB groups (15.9–16.4%). Overall, the research panel used in the study was highly heterogeneous and contrasting, especially for vegetative period and grain yield.

### 2.2. Adjustment of Grain Element Concentration

The correlation analysis was conducted to find the relationship between the concentrations of the 15 elements and agronomic traits (grain yield, protein content, and TKW) (Table 1). The analysis was performed separately for each year. Protein content was found to be positively correlated with the concentrations of 11 of the elements in one or both years. Only Ca, K, Co, and Rb were not correlated with protein content. However, the correlation coefficients between protein content and the elements ranged from 0.2 to 0.4, indicating weak relationships. Mg concentration was significantly correlated with 10 of the elements, with correlation coefficients exceeding 0.6 for P, S, Cu, and Mn. P and S concentrations were also positively correlated with microelements (Cu, Mn, and Zn) and toxic trace elements (Cd, Ni, and Mo). Among the macroelements, variation in Ca and K concentrations was the least correlated with any agronomic trait or other element. Co, Rb, and Fe (2018) concentrations were not correlated with any trait.

All significant correlations presented in Table 1 were used for adjustment of element concentrations through multiple regression. The basic statistical parameters of the original and adjusted values are presented in Table 2. The adjusted data had the same means, but the differences between the minimum and maximum values were reduced. This resulted in the reduction of the coefficient of variation (CV) for all elements by 0.2–6.4%. A factorial ANOVA (genotype x year) was performed for each element, using original and adjusted data (Table S2). The level of significance of the main factors (genotype and year), using original and adjusted values, was identical. However, for five of the elements (Ca, Mg, P, S, and Cu) the interaction of genotypes x year was not significant (*p* > 0.05) using the original values, although it was significant when adjusted values were used in the ANOVA. This indicates that adjustment contributed to the higher capacity to distinguish significance of these important interactions. Overall, the adjustment of element concentrations using multiple regression was well justified and provided a more balanced approach for comparison of genetic resources and individual genotypes.

The broad sense H^2^ was calculated for each year using original and adjusted values (Table 2). The adjustment slightly increased H^2^ for Mg and P and decreased for S and Cu, in both years. The average H^2^ across all elements was 0.59 for the original data and 0.58 for the adjusted data. The H^2^ calculated based on a factorial ANOVA was similar for K, Mg, P, Cu, Mn, Ni, and Mo for original and adjusted values. Slightly different H^2^ estimates were found for Ca (0.73 for the original data vs. 0.62 for the adjusted), S (0.81 vs. 0.62), Fe (0.58 vs. 0.47), Zn (0.27 vs. 0.37), Cd (0.76 vs. 0.67), and Sr (0.87 vs. 0.77).

### 2.3. Variation for Element Concentrations

Phosphorus had the highest concentration of all the elements in wheat grain, at 5180 and 4699 µg/g in 2017 and 2018, respectively (Table 2). The other macroelement concentrations (µg/g) in decreasing order were K (3642–3652), followed by S (2050–2059), Mg (1208–1225), and Ca (362–397). The variation between years was limited for K, S, and Mg, but exceeded 10% for P and Ca. The ranking of the macroelements for grain concentration coefficient of phenotypic variation, based on the original values, was Ca (14.4%), followed by K (12.9%), P (11.3%), S (9.5%), and Mg (8.8%). Among the microelements, Zn had the highest concentration (µg/g) in the grain (48.8–53.3 over the two years), followed by Mn (43.1–44.8), Fe (35.8–37.7), and Cu (3.72–4.66). The ranking of the microelements for grain concentration coefficient of phenotypic variation, based on the original values, was Zn (17.3%) followed by Cu (14.5%), Fe (13.6%), and Mn (11.5%).

Among the three toxic trace elements, Ni had the highest concentrations, at 0.212 and 0.148 µg/g in 2017 and 2018, respectively. Ni was also a highly variable element, both within and between years, with CV exceeding 23.6%. Cd and Co had only low concentrations (<0.044 µg/g) that were highly variable (CV 17.1–35.8%). The three remaining trace elements (Mo, Rb, and Sr) were also characterized by low concentrations (0.31–4.35 µg/g) and high variability (CV 18.9–30.3%).

### 2.4. Elements’ Suitability for Genetic Enhancement

The progress for wheat genetic enhancement to increase or decrease the concentration of a certain element depends on several factors: the degree of phenotypic variation within the germplasm, the share of the genetic component in this variation, as well as the dependence of the element concentration on other traits. Table 3 summarizes three main criteria characterizing suitability of elements for genetic enhancement. The coefficient of phenotypic variation varied from 8.8% (Mg) to 35.7% (Cd). It is assumed that higher variation provides the opportunity for selection. Therefore, the element with the highest variation was ranked 1 and the lowest, 15. H^2^ calculated based on a factorial ANOVA (genotype x year) of the original data varied from 0.27 (Zn) to 0.87 (Sr). Similar to CV, the highest value of H^2^ was ranked 1 and the lowest, 15. The ideal breeding trait may have a variation independent of other traits.

For each element, Table 3 provides the number of significant correlation coefficients with three agronomic traits (grain yield, protein content, and TKW) and macroelements, based on the data in Table 1. The concentrations of Co and Rb did not correlate with any trait or element. They were ranked the highest (rank 1.5), while Cu, Zn, and Cd had 10 significant correlations, being ranked the lowest (rank 14). The last column in Table 3 presents the sum of three ranks, which were again ranked from lowest (better suited for genetic enhancement) to highest. Three trace elements (Sr, Mo, and Co) were the highest ranked, representing breeding traits that are relatively easy to improve through breeding. These were followed by Ca, Cd, Rb, and K. The important biofortification elements Fe and Cu were ranked 9 and 10, respectively. Mn and Zn were among the lowest ranked, representing traits that are difficult to improve genetically.

### 2.5. Genetic Resources Characterization for Grain Ionome

**Figure 2:**
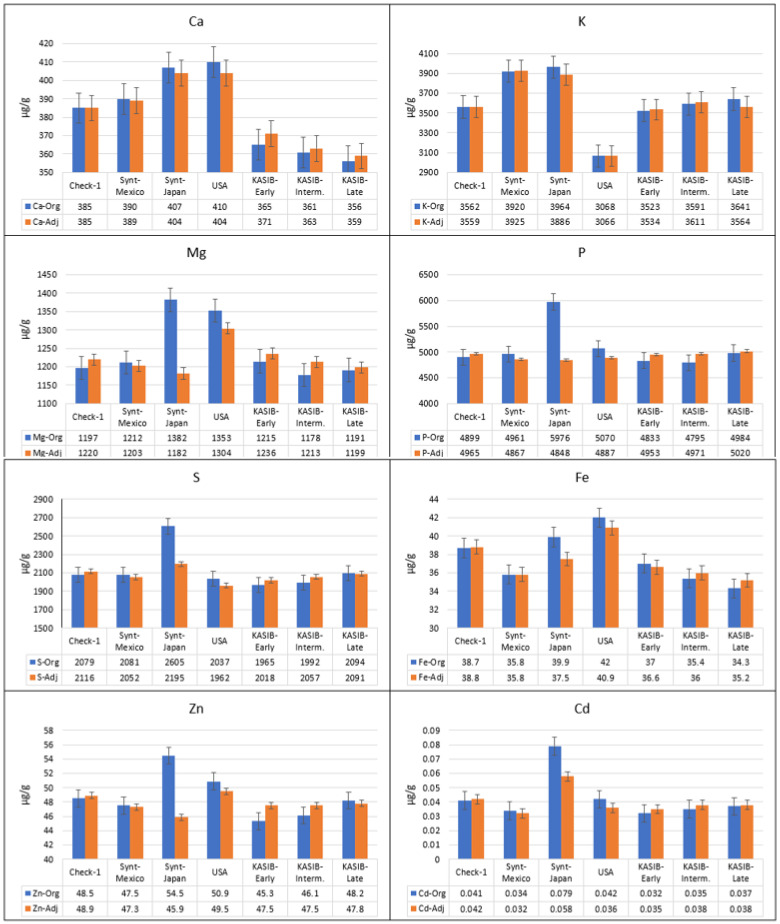
Average grain element concentrations for the genetic resource groups across 2017 and 2018 (blue, original values, and orange, adjusted values), bars represent standard error.

The original and adjusted average concentrations across 2017 and 2018 for different groups of genetic resources for the macroelements Fe, Zn, and Cd, are presented in Figure 2, and for all elements for each year independently in Table S3. There is a clear difference between the original and adjusted values. For Mg, P, S, and Zn, the Japanese synthetics were clearly superior when the original data were used. However, the adjusted means demonstrated different performance, comparable to other groups of genetic resources.

Taking into account the adjusted values, the CIMMYT and Japanese synthetics had significantly higher concentrations of K (9.2–10.1% higher) and Sr (9.4–16.4% higher), compared to local Check-1 (Pamyati Azieva). The Japanese synthetics also had the highest concentrations of Ca (4.9% higher, compared to local Check-1), S (3.7%), Cd (38.1%), and Mo (4.7%). The US cultivars had concentrations of Ca as high as the Japanese synthetics, and the highest concentrations of Mg (6.9% higher than Check-1) and Fe (5.4% higher). This group was also characterized by low concentrations of K (13.8% lower, compared to Check-1) and Mo 11.8% lower. The KASIB germplasm had near-average values for most elements. However, there were differences between the early, intermediate, and late maturity groups. For Mg and Fe, there were decreasing concentrations from early to intermediate to late material of 1.5–2%. For S and Zn, concentrations increased in the later-maturing germplasm, also by 1.5–2%.

### 2.6. Superior Germplasm

All of the germplasm was ranked for concentration of each element, based on mean adjusted values for 2017–2018 (Table S4). Superior genotype performance was defined as being in the top 15 entries, based on the highest concentration of macro- and microelements. For the trace elements, including toxic metals, favorable performance was considered as being in the bottom 15 entries, based on the lowest concentrations. The germplasm was also ranked for grain yield and protein content. Table 4 presents the 10 highest-yielding genotypes and 20 entries with favorable concentrations for at least three elements. The five highest-yielding genotypes had high concentrations of Ca, including cv. Silach, with a high concentration of Mg and low Rb; Element 22, with high concentrations of P and S, but low Ni; and breeding line Lutestsens 1296, with high K and low Cd concentrations. The second-highest yielding genotype Lutestsens 15-14 was characterized by high concentrations of the essential microelements Mn and Zn. As expected, all top-yielding genotypes belonged to the KASIB group of germplasm.

The genotype with favorable concentrations of six elements was Ukr-Od 1530.94/*Aegilops squarrosa*(392) (high K, P, S, and Zn, and low Cd and Mo). Superior performance for five elements was found for Lutestsens 1103 (Ca, Mg, Mn, Ni, and Mo), Freyr, USA (Mg, Zn, Ni, Mo, and Rb), and Aisberg/*Ae. squarrosa*(369)//Demir (Cu, Zn, Cd, Co, and Mo). High Fe and Zn were found for cvs Stepnaya 253 and Alpine. Several genotypes demonstrated low concentrations of at least two trace elements: Novosibirskaya 41 (Cd and Ni), Lutestsens 1103 (Ni and Mo), Lutestsens 248-01 (Mo and Sr), Lutestsens 15-12 and Langdon/IG 131606 (Co and Ni), Freyr (Ni, Mo, and Rb), Aisberg/*Ae. squarrosa*(369)//Demir and Ukr-Od 952.92/*Ae. squarrosa*(1031) (Cd, Co, and Rb), Ukr-Od 1530.94/*Ae. squarrosa*(458) (Cd and Co), Ukr-Od 1530.94/*Ae. squarrosa*(392) (Cd and Mo), and Ukr-Od 1530.94/*Ae. squarrosa*(458) (Cd and Rb). Overall, there was a diversity of germplasm with high macro- and microelement concentrations and low trace element concentrations within the material studied. The superior genotypes were found in all groups of material, including KASIB, US cultivars, and primary synthetics.

## 3. Discussion

The growing concern and interest in healthy food, as well as the strategies to combat malnutrition, have resulted in a greater research and development priority to improve the nutritional quality of wheat grain [17]. Recent reviews summarized the achievements and challenges of biofortification for essential elements, including Cu, Fe, Se, and Zn [18], Zn [19], Fe [20], and Se [21]. There is general agreement that agronomic approaches and genetic improvement need to be combined to achieve the best results and to raise the concentrations to target levels. There is evidence from experiments with Fe [22] and Zn [6] that biofortified foods have significantly improved in nutritional value in human diets. The concept of biofortification of food crops or cereals has yet to attract sufficient attention in Russia, either in research or in the plant-breeding community. Recent reviews by Loskutov and Khlestkina [23] and Shelenga et al. [24], and the study of Bityutskii [7] demonstrated that there is an understanding of the importance of the nutritional value of wheat, barley, and oats, taking into consideration concentrations of essential microelements. However, this has not yet been converted into integrated biofortification programs for wheat and other staple crops.

Two wheat-grain ionome studies recently conducted in Russia and Kazakhstan by our research group provided important information on the element composition of wheat grain and laid the foundation for the development of targeted programs to optimize the concentration of macro- and microelements, and trace metals. Abugalieva et al. [25] evaluated the concentrations of macroelements, toxic heavy metals, and microelements in 179 wheat grain samples collected in 2017 and 2018 from production fields across northern Kazakhstan and the Omsk region of Russia. The concentrations of essential microelements were similar to wheat grain produced in other countries, with exception of Zn. The concentrations of this important element in Omsk and East Kazakhstan were 50 µg/g above the values targeted by the Harvest Plus biofortification program. Even with the losses of Zn during milling, the grain from these regions could be particularly beneficial for human health. In the second study, a KASIB trial from six locations in Kazakhstan and Russia in 2017–2018 was used for grain ionomics analysis to evaluate genotype × environment interaction [8], as the effect of year was the least important. For several elements (P, S, Cu, Mn, and Mo), the effect of the site was 2–3 times higher, compared to the effect of the genotype. The effects of the genotype and site were similar for Ca, Mg, Fe, Cd, and Sr concentrations. That study recommended establishment of a modern biofortification breeding program using phenotyping and genomic tools, and effectively using the multilocational KASIB network.

The current ionomics study is the third in this series addressing the specific issue of variation of element concentrations in diverse spring wheat genetic resources, evaluated over two years under a typical production system in Western Siberia. The study revealed the advantage of the ionomics approach, when all important grain macro- and microelements, and trace metals are phenotyped to analyze their relationships and to evaluate germplasm in an integrative manner. This study proved the importance of protein content as a key trait affecting the concentration of almost all grain elements, reported previously in Fatyukha et al. [26]. Macroelements Mg, P, and S were also significantly correlated with other elements. The share of the seed-coat increases in smaller grains normally results in higher protein content and some elements’ concentrations. However, this relationship was not well pronounced in this study. The grain yield in this diverse germplasm varied fourfold between the different groups, and this trait also significantly correlated with the concentrations of a number of elements. Considering the diversity of the germplasm and the high variation for agronomic traits, the concentrations of all elements were adjusted based on the correlations. Similar adjustments were made by Fatyukha et al. [26], using protein content and P concentration as variables. The adjustments made in this study were well justified and allowed more precise evaluation of genetic resources.

Primary synthetic wheat developed from crosses of durum wheat with *Ae. tauschii* has been reported as a source of high concentrations of microelements, including Fe and Zn [27,28]. In the current study, based on original values, primary synthetics from Japan also demonstrated high concentrations of a number of elements, including Ca, Mg, P, S, Fe, and Zn. However, after adjustment using multiple regression, the synthetic wheat germplasm largely lost its advantage. Superior germplasm combining high concentrations of macro- and microelements, and low concentrations of trace elements was identified in all germplasm groups, including the KASIB material, the US cultivars, and both synthetics groups. The genotypes with favorable concentrations of five and six elements were also identified in all germplasm groups. Previous study of KASIB trials across six sites [8] included around 40 entries, which were also included in the present study. Cvs Silach and Novosibirskaya 41, identified as superior for agronomic and ionomic traits in a multilocational trial, also demonstrated superior performance in this study, validating the earlier results.

The crossing strategy to incorporate and combine optimal concentrations of a wide range of elements depends on the nature of the germplasm. Synthetic wheat with a low yield and a number of undesirable traits, such as spike threshability, requires a top-and-back crossing scheme to transfer useful traits, while maintaining and improving grain yield. Several synthetics from the current study possess resistance to leaf, stem rust, and powdery mildew [29], making them attractive as parental material. Disease resistance, short stature, and earliness were additional positive traits of the US cultivars for improvement of Siberian wheat for the ionome profile. Simple crosses and development of a large population may be sufficient to combine positive traits of KASIB and US materials. However, the back-and-top crosses with local material may also be efficiently used. A crossing program within the KASIB breeding network would be straightforward, based on simple crosses and consequent selection.

Genetic gains in utilization of genetic resources for practical breeding will largely depend on the traits’ heritability and phenotyping precision. The KASIB multilocational study established the broad-sense heritability values for macroelements: Mg (0.59) > Ca (0.50) > K (0.44) > P (0.30) > S (0.20), and for microelements: Zn (0.44) > Mn (0.41) > Cu (0.40) > Fe (0.38) [8]. In the current study, the H^2^ values were higher, due to the utilization of one location, and different for macroelements: S (0.81) > Ca (0.73) > Mg (0.67) > K (0.61) > P (0.60) and microelements: Cu (0.61) > Fe (0.58) > Mn (0.55) > Zn (0.27). This difference is explained by the diversity of the material used and the testing site’s genotype x environment interaction. However, for trace elements, the ranking in the two studies was almost identical. Considering H^2^, the coefficient of phenotypic variation, and correlations with other traits, trace elements seem to be easier ionomic targets for genomic enhancement. At the same time, important elements, such as Zn, Fe, and Cd, are more influenced by environment and relations with other traits. Consequently, their improvement requires high throughput and precision phenotyping. Determination of grain protein content as a key variable affecting element concentrations will be an important component of a successful selection program.

The levels of heritability for elements varied from low to high, suggesting that breeding improvement for certain elements, such as Sr, could be more effective, compared to Zn. Further, there were positive correlations between certain elements. Positively correlated traits with high heritability could be improved more efficiently by selecting for only one of the correlated traits. For improving elements with low heritability, low selection pressure would be effective. For the elements with no, low, or negative correlations, some kind of intuitive index selection could be used that would allow balancing moderate defects in one trait with obvious gain in others [30] (Simmonds, 1981).

The utilization of genomics greatly enhances breeding efficiency and genetic gains [31]. The material used in this study was subjected to a GWAS analysis, and a number of marker–trait associations were identified for agronomic traits, including protein content [16]. However, grain element composition was not included in that analysis. CIMMYT synthetic wheat used in the current study was assayed for concentrations of 10 minerals in the USA as part of a larger synthetics panel [32]. Multitraits and stable marker–trait associations were identified, and the 13 top synthetic lines were recommended for higher concentrations of beneficial grain minerals (Cu, Fe, Mg, Mn, Ni, and Zn). The next logical step following the current study is to evaluate the effects of functional markers and undertake a GWAS analysis to identify marker–trait associations, validate them against previously published data, and make recommendations for use in practical breeding.

Grain element composition is affected by molecular homeostasis, physiological and biochemical alterations, and intracellular compartmentalization [33]. The current study did not attempt to analyze physiological and biochemical pathways affecting concentrations of elements in wheat grain. However, wheat physiology is an important component of an integrated program to increase the concentrations of beneficial elements and decrease the amounts of toxic metals in the grain. The current ionome study contributes to the development of wheat biofortification programs in Russia to develop healthy grain for domestic and international markets.

## 4. Materials and Methods

### 4.1. Panel Composition and Evaluation

The panel in 2017 and 2018 comprised 135 entries, including two checks, as listed in Table S5. The research material included 37 primary synthetics from CIMMYT, developed through crosses between Ukrainian winter durum wheat cultivars and several accessions of *Aegilops tauschii* from its gene bank. The development of the synthetics through targeted selection under abiotic and biotic stresses was described by Morgounov et al. [34]. Eight primary synthetics developed by Kyoto University in Japan [35] comprised the second group. The US cultivars (14 in total) included hard red spring wheat entries, primarily from the University of Minnesota and from Syngenta. Material from KASIB was represented by new cultivars and breeding lines, and was divided into early maturing (15 entries), intermediate (42 entries), and late-maturing (17 entries) groups. The two checks were widely grown spring wheat cultivars in the Omsk region, Pamyati Azieva (Check-1) and Serebristaya (Check-2), representing early and intermediate maturity groups, respectively. The main contributors of the KASIB germplasm were Omsk State Agrarian University with 17 entries, and Omsk Agrarian Research Center with 14 entries. The panel also included 17 cultivars and breeding lines from Kazakhstan.

The trial was planted in the experimental field of Omsk State Agrarian University (55.0404° N; 73.3604° E) as a randomized complete block design, with plots of 1 m^2^ and four replicates. The soil of the experimental field was meadow chernozem, with 5% organic matter content and an average availability of NPK. The preceding crop was black fallow. Spring soil preparation comprised harrowing in early May, followed by shallow cultivation and harrowing in mid-May. Planting took place between May 15 and May 20, in both years. The trials were harvested in the first week of September. Neither fertilizer nor fungicides were applied. Weeds were controlled by the application of common herbicide after the tillering stage in mid-June. The field observations included agronomic traits, including heading dates, disease evaluations, yield, and yield components. The CIMMYT Wheat Physiology Manual [36] was used as a guide for germplasm evaluations for all traits and diseases. The protein content in the grain was determined using Infratec FOSS 1841.

### 4.2. Grain Ionomics Analysis

The grain sample (10 gr) for analysis was taken after manual harvesting of the plots and machine threshing. The sample was random from a bag of threshed and cleaned seeds. One sample was taken from each replication. All the collected samples were shipped to the UK and stored in a dry (<60% humidity) and cool (18–20 °C) storage facility. The samples were once again cleaned prior to ionomics analysis and the grain moisture content was determined. The ionomic analyses were performed with a phenotyping platform at the University of Nottingham (UK) through the European Plant Phenotyping Network, a research infrastructure project funded by the Horizon 2020 Program of the EU, which offered researchers access to a wide range of state-of-the-art plant-phenotyping facilities. The analyses were performed using a state-of-the-art PerkinElmer NexION 2000 inductively coupled plasma mass spectrometer (ICP-MS).

The samples were prepared for the ICP-MS analysis in the adjoining high-throughput preparation laboratory. Wheat grains were transferred into the Pyrex test tubes, weighted, and initially predigested with 1 mL concentrated trace-metal-grade nitric acid Primar Plus (Fisher Chemicals, Hampton, VA, USA) spiked with 20 μg/L of indium internal standard, for approximately 20 h at room temperature. Indium was added to the nitric acid as an internal standard for assessing errors in dilution, variations in sample introduction, and plasma stability in the ICP-MS instrument. After the predigestion step, samples were transferred into DigiPREP MS dry block heaters (SCP Science, QC, Canada; QMX Laboratories, Thaxted, UK) and digested for 4 h at 115 °C. After cooling down, 1 mL of trace-metal-grade hydrogen peroxide (Primar, Fisher Chemicals, Hampton, VA, USA) was added to the tubes, and samples were digested in the dry block heaters for an additional 2 h at 115 °C, and then diluted to 10 mL with 18.2 MΩcm Milli-Q Direct water (Merck Millipore, Burlington, MA, USA).

Five replicate analyses were conducted for each sample from each replication, and the mean value represented the sample’s final readings. The ionomics results were obtained for 23 elements: macroelements, Ca, K, Mg, P, and S; microelements, B, Fe, Cu, Na, Mn, and Zn; toxic trace elements, As, Cd, Co, Cr, Ni, Pb, and Se; and trace elements, Li, Mo, Rb, and Sr. The concentrations were either at trace levels or below the limit of quantification for B, Na, As, Cr, Pb, Se, Li, and Ti. Therefore, these elements were excluded from the analyses. For the remaining 15 elements, all of the concentrations were normalized to the weight of the samples and expressed as µg/g of dry weight.

### 4.3. Data Processing and Statistical Analysis

Correlations between individual element concentrations and the other variables, viz., grain yield, 1000 kernel weight (TKW), protein content, and macroelement concentrations, were calculated using Microsoft Excel. The correlation analysis results (Table 1) were used to adjust the original element concentration values using multiple regression on the following traits: grain yield, protein content, TKW, and concentrations of Ca, K. Mg, P, and S. The concentration of each element was adjusted only for the traits with significant correlations. Some elements (Co and Rb) did not correlate with any variable and, therefore, no adjustments were made. Some elements (Ca, K, and Fe) did not correlate with other traits in one year but correlated in another year, and adjustments were made only for the year with significant correlations. For all other elements, adjustments were made in both years and the number of variables in the regression varied from one to five.

A factorial ANOVA (genotype x year) was used for statistical analysis for all agronomic traits and for each element independently, using R version 3.4 [37]. Broad-sense heritability (H^2^) was estimated for each element in individual trials (for each year separately) based on the ANOVA results. All analyses were conducted separately for the original and adjusted values.

## Figures and Tables

**Table 1:** Correlation coefficients between agronomic traits and element concentrations in grain, 2017–2018.

Element	Year	Yield	PC	TKW	Ca	K	Mg	P	S
Ca	2017	−0.11	0.06	−0.14	-	0.16	0.15	0.01	0.09
	2018	−0.14	0.06	−0.15	-	−0.03	0.22 *	0.03	0.11
K	2017	−0.13	0.03	0.10	0.16	-	−0.07	−0.07	0.30 *
	2018	−0.19	0.16	0.17	−0.03	-	0.07	0.07	0.09
Mg	2017	−0.24 *	0.43 *	−0.07	0.15	−0.07	-	0.79 *	0.60 *
	2018	−0.19	0.29 *	−0.06	0.22 *	0.07	-	0.76 *	0.55 *
P	2017	−0.14	0.32 *	0.08	0.01	0.20 *	0.79 *	-	0.68 *
	2018	−0.22 *	0.30 *	0.05	0.03	0.48 *	0.76 *	-	0.20 *
S	2017	−0.23 *	0.45 *	0.13	0.09	0.30 *	0.60 *	0.68 *	-
	2018	−0.34 *	0.47 *	0.12	0.11	0.09	0.55 *	0.20 *	-
Cu	2017	−0.14	0.28 *	−0.02	−0.03	0.10	0.62 *	0.68 *	0.54 *
	2018	−0.21 *	0.25 *	0.01	0.08	0.21 *	0.60 *	0.60 *	0.47 *
Fe	2017	−0.10	0.23 *	0.29 *	−0.01	−0.14	0.23 *	−0.06	−0.09
	2018	−0.13	0.17	−0.14	−0.03	−0.12	0.05	−0.04	−0.10
Mn	2017	−0.13	0.35 *	−0.02	0.11	−0.10	0.68 *	0.57 *	0.49 *
	2018	−0.09	0.05	0.09	0.22 *	−0.10	0.63 *	0.46 *	0.38 *
Zn	2017	−0.03	0.20 *	0.04	−0.08	0.02	0.53 *	0.73 *	0.48 *
	2018	−0.30 *	0.26 *	0.02	0.09	0.27 *	0.49 *	0.63 *	0.50 *
Cd	2017	−0.20 *	0.29 *	0.01	0.00	−0.02	0.46 *	0.44 *	0.49 *
	2018	−0.22 *	0.38 *	0.10	0.09	0.05	0.37 *	0.32 *	0.40 *
Co	2017	0.01	0.07	−0.19	0.12	0.14	0.17	0.14	0.04
	2018	0.01	0.05	0.04	0.19	0.12	0.11	0.15	0.15
Ni	2017	−0.08	0.15	−0.01	−0.01	0.09	0.25 *	0.25 *	0.23 *
	2018	−0.24 *	0.36 *	0.01	−0.03	0.14	0.23 *	0.28 *	0.31 *
Mo	2017	−0.07	0.20 *	0.05	0.01	0.17	0.12	0.20 *	0.24 *
	2018	−0.02	0.15	0.18	0.05	0.27 *	0.21 *	0.33 *	0.26 *
Rb	2017	−0.05	0.06	0.16	−0.02	0.12	0.04	0.08	0.07
	2018	−0.18	0.12	0.01	0.07	0.18	−0.01	0.00	0.16
Sr	2017	−0.34 *	0.22 *	−0,08	0.60 *	0.11	0.17	−0.00	0.16
	2018	−0.34 *	0.31 *	−0.22 *	0.65 *	0.04	0.17	0.04	0.16
Number of significant correlations		11	18	3	4	6	16	14	15

*—significant at *p* > 0.05.

**Table 2:** Statistical parameters for original and adjusted values across all germplasm in 2017–18.

Element-Year	Data	Adjustment Variables ^1^	Mean	Min	Max	CV, %	H^2^	r (Original–Adjusted)
Macroelements								
Ca-2017	Original	-	362	237	518	15.1	0.59	-
Ca-2018	Original	-	397	266	531	13.8	0.49	0.98
	Adjusted	Mg		273	523	13.6	0.50	
K-2017	Original	-	3652	2654	5453	10.2	0.67	0.99
	Adjusted	S		2715	5314	10.1	0.69	
K-2018	Original	-	3642	2298	4992	15.7	0.79	-
Mg-2017	Original	-	1208	922	1532	9.1	0.60	0.56
	Adjusted	YLD, PC, P, S		997	1400	5.4	0.62	
Mg-2018	Original	-	1225	926	1524	8.5	0.58	0.66
	Adjusted	PC, Ca, P, S		1007	1461	5.6	0.69	
P-2017	Original	-	5180	3906	6701	10.2	0.43	0.54
	Adjusted	PC, K, Mg, S		4401	5995	5.6	0.62	
P-2018	Original	-	4699	3428	6993	12.3	0.57	0.48
	Adjusted	YLD, PC, K, Mg, S		3835	5500	5.8	0.68	
S-2017	Original	-	2059	1710	2682	9.5	0.69	0.60
	Adjusted	YLD, PC, K, Mg, P		1768	2386	5.8	0.59	
S-2018	Original	-	2050	1572	2698	9.6	0.71	0.64
	Adjusted	YLD, PC, Mg, P		1762	2433	6.1	0.66	
Microelements								
Cu-2017	Original	-	4.66	3.09	7.08	13.8	0.54	0.76
	Adjusted	PC, Mg, P, S		3.53	6.29	10.4	0.63	
Cu-2018	Original	-	3.72	2.30	6.06	15.3	0.57	0.74
	Adjusted	YLD, PC, Mg, P, S		2.67	5.22	11.3	0.55	
Fe-2017	Original	-	37.7	21.1	53.6	14.2	0.50	0.90
	Adjusted	PC, Mg		21.4	51.8	12.9	0.40	
Fe-2018	Original	-	35.8	22.5	47.2	12.9	0.25	-
Mn-2017	Original	-	43.1	31.9	60.0	11.2	0.58	0.72
	Adjusted	PC, Mg, P, S		31.0	52.4	8.2	0.58	
Mn-2018	Original	-	44.8	31.9	59.1	11.7	0.63	0.81
	Adjusted	Mg, P, S		32.6	52.4	8,2	0.70	
Zn-2017	Original	-	53.3	31.1	77.7	15.6	0.16	0.74
	Adjusted	PC, Mg, P, S		40.0	69.3	10.9	0.27	
Zn-2018	Original	-	41.8	23.9	76.3	19.1	0.42	0.67
	Adjusted	PC, K, Mg, P, S		26.4	62.0	13.1	0.39	
Toxic trace elements								
Cd-2017	Original	-	0.044	0.019	0.117	35.8	0.66	0.75
	Adjusted	YLD, PC, Mg, P, S		0.024	0.099	27.7	0.57	
Cd-2018	Original	-	0.031	0.011	0.088	35.6	0.68	0.77
	Adjusted	YLD, PC, Mg, P, S		0.013	0.071	27.7	0.55	
Co-2017	Original	-	0.010	0.006	0.021	27.0	0.72	-
Co-2018	Original	-	0.009	0.006	0.017	17.1	0.27	-
Ni-2017	Original	-	0.212	0.113	0.356	23.6	0.44	0.94
	Adjusted	Mg, P, S		0.113	0.360	22.4	0.44	
Ni-2018	Original	-	0.148	0.078	0.312	26.6	0.57	0.85
	Adjusted	YLD, PC, Mg, P, S		0.075	0.224	22.6	0.47	
Trace elements								
Mo-2017	Original	-	0.347	0.209	0.579	22.4	0.71	0.94
	Adjusted	PC, P, S		0.208	0.554	22.6	0.69	
Mo-2018	Original	-	0.308	0.195	0.592	24.0	0.78	0.91
	Adjusted	K, Mg, P, S		0.183	0.554	21.4	0.74	
Rb-2017	Original	-	4.35	2.29	6.75	21.7	0.25	-
Rb-2018	Original	-	3.51	2.20	5.34	18.9	0.25	-
Sr-2017	Original	-	2.09	0.914	3.627	30.3	0.81	0.91
	Adjusted	YLD, PC, TKW		0.909	3628	27.7	0.77	
Sr-2018	Original	-	2.42	1.175	4.307	27.2	0.75	0.82
	Adjusted	YLD, PC, TKW		1.366	4.053	22.2	0.63	

^1^ YLD, yield; PC, protein content; TKW, 1000 kernel weight.

**Table 3:** Parameters characterizing elements’ suitability for genetic enhancement.

Element	Coefficient of Phenotypic Variation for 2017–2018 Means		Number of Significant Correlations with Agronomic Traits and Other Elements		H^2^ Based on ANOVA of Original Data		Overall Sum of Ranks	
	%	Rank	Value	Rank	Value	Rank	Value	Rank
Ca	14.5	9	1	3.5	0.73	5	17.5	4
K	13.0	11	1	3.5	0.61	8	22.5	7
Mg	8.8	15	8	9.5	0.67	6	30.5	12
P	11.3	13	9	12.5	0.60	10	34.5	14
S	9.6	14	9	12.5	0.81	3	28.5	11
Cu	14.6	8	10	11	0.61	9	28.0	10
Fe	13.6	10	2	5	0.58	11	26.0	9
Mn	11.5	12	7	7	0.55	12	31.0	13
Zn	17.4	7	10	14	0.27	15	36.0	15
Cd	35.7	1	10	14	0.76	4	19.0	5
Co	22.1	5	0	1.5	0.65	7	13.5	3
Ni	25.1	3	8	9.5	0.49	13	25.5	8
Mo	23.2	4	7	7	0.84	2	13.0	2
Rb	20.3	6	0	1.5	0.48	14	21.5	6
Sr	28.8	2	7	7	0.87	1	10.0	1

**Table 4:** Genotypes with the highest grain yield, highest concentrations of macro- and microelements, and lowest concentrations of trace elements.

Entry	Genotype	Group *	Highest/Lowest Elements	Grain Yield		Protein Content	
				g/m^2^	Rank	%	Rank
-	Pamyati Azieva (Check-1)		-	399	-	16.5	-
-	Serebristaya (Check-2)		-	471	-	14.9	-
151	Lutestsens KS 963	K-E	Ca, Mg	572	1	17.2	66
112	Lutestsens 15-14	K-L	Mn, Zn	557	2	16.5	101
91	Lutestsens 7-04-4	K-I	-	541	3	17.5	54
164	Silach	K-L	Ca, Mg, Rb	541	4	16.7	95
94	Element 22	K-L	Ca, P, S, Ni	535	5	17.0	75
152	Lutestsens 1296	K-I	Ca, K, Cd	521	6	15.6	125
157	OmGAU-100	K-L	Ca, P, Cu	518	7	16.4	103
116	Uralosibirskaya	K-I	-	515	8	17.6	52
85	Lutescens 310-00-1	K-I	P, Rb	514	9	17.5	54
89	Aestivum 947	K-I	Ni	514	10	15.8	119
156	Novosibirskaya 41	K-E	P, Cu, Cd, Ni	482	23	19.0	16
143	Lutestsens 1103	K-I	Ca, Mg, Mn, Ni, Mo	469	30	16.1	111
125	Stepnaya 253	K-I	Ca, Fe, Zn	468	31	14.1	133
114	OmGAU-90	K-I	Ca, Mg, S	466	33	15.7	121
132	Lutestsens 248-01	K-I	Mn, Zn, Mo, Sr	394	58	15.9	117
66	RBOT	USA	Cu, Fe, Rb	386	63	18.4	28
87	L 485	K-E	Ca, P, Mn	378	66	17.0	73
103	Lutestsens 15-12	K-I	Cu, Co, Ni	372	71	17.6	51
73	Freyr	USA	Mg, Zn, Ni, Mo, Rb	354	75	19.0	15
61	Pandur/*Ae. Squarrosa*(409)	S-M	K, S, Cu, Co	330	79	17.4	57
78	Alpine	USA	Mg, Fe, Zn, Rb	321	81	17.9	43
13	Ukr-Od 1530.94/*Ae. Squarrosa*(392)	S-M	Fe, Mn, Sr	313	82	17.2	71
77	Brennan	USA	Mg, Fe, Mn	297	87	18.7	19
36	Aisberg/Ae. Squarrosa (369)//Demir	S-M	Cu, Zn, Cd, Co, Mo	273	94	16.3	107
3	Ukr-Od 952.92/*Ae. Squarrosa*(1031)	S-M	Cd, Co, Rb	255	100	17.0	76
12	Aisberg/*Ae. Squarrosa*(511)	S-M	Ca, S, Cu, Mo	245	103	16.8	87
6	Ukr-Od 1530.94/*Ae. Squarrosa*(458)	S-M	P, Cd, Co	213	115	17.7	47
57	Ukr-Od 1530.94/*Ae. Squarrosa*(392)	S-M	K, P, S, Zn, Cd, Mo	209	116	18.2	34
16	Ukr-Od 1530.94/*Ae. Squarrosa*(458)	S-M	Ca, Cd, Rb	206	118	17.2	69
22	Langdon/IG 48042	S-J	Ca, S, Fe	141	125	20.7	6
51	Langdon/IG 131606	S-J	Ca, Co, Ni	97	130	20.6	7
	LSD 0.05			15	-	0.3	-

* K—KASIB group; E—early; I—intermediate; L—late; S—synthetics; M—Mexico; J—Japan.

## Data Availability

The phenotypic data are available on request from the corresponding author.

## Supplementary Materials

The following supporting information can be downloaded at: https://www.mdpi.com/article/10.3390/plants11020149/s1, Table S1: ANOVA F-value probability of the effects of genotypes, year, and their interactions for days to heading, grain yield, protein content, TKW, and element composition, Table S2: Variation for agronomic traits and elemental grain concentration in genetic resource groups in 2017–18, Table S3: Variation for agronomic traits and elemental grain concentration in genetic resource groups in 2017–18, Table S4: Variation in grain yield, protein content, 1000 kernel weight, and elemental composition of different groups of germplasm, Omsk, 2017–2018, Table S5: Wheat genotypes used in the study.
